# Determinants of cervical cancer screening uptake among reproductive-age women in southwest Ethiopia: a case-control study

**DOI:** 10.3389/fonc.2024.1424810

**Published:** 2024-10-23

**Authors:** Tewodros Yosef, Bitewlgn Birhanu, Nigusie Shifera, Bayu Begashaw Bekele, Adane Asefa

**Affiliations:** ^1^ School of Public Health, College of Medicine and Health Sciences, Mizan-Tepi University, Mizan-Teferi, Ethiopia; ^2^ School of Medicine, Faculty of Health, Deakin University, Waurn Ponds, VIC, Australia; ^3^ Division of Public Health Sciences, Department of Surgery, Washington University School of Medicine, St Louis, MO, United States

**Keywords:** cervical cancer, screening uptake, reproductive-age women, knowledge, attitude, Ethiopia

## Abstract

**Background:**

Cervical cancer is a major global health issue, with 604,000 diagnoses and 342,000 deaths in 2020. Despite the importance of early detection, only 5% of eligible women in Ethiopia are screened. Therefore, this study aimed to assess the determinants of cervical cancer screening uptake among reproductive-age women at selected public hospitals in southwest Ethiopia.

**Methods:**

A case-control study involving 392 women (98 cases and 294 controls) aged 15-49 was conducted across three hospitals. Cases were women aged 15 to 49 who had cervical cancer screening, while controls were reproductive-age women seeking antenatal care or family planning but not screened. Data were collected via face-to-face interviews with pretested questionnaires and analyzed using SPSS 25. Bivariate analysis identified candidate variables with P-values < 0.25, and a multivariable logistic regression model determined factors with P-values < 0.05 as significant for cervical cancer screening uptake.

**Results:**

Determinants of cervical cancer screening uptake included high knowledge of screening (AOR=6.23; 95%CI: 1.96, 19.79), a positive attitude toward screening (AOR=6.12; 95%CI: 2.40, 15.58), women aged 30-39 (AOR=3.94; 95%CI: 1.79, 8.63) and 40-49 (AOR=3.54; 95%CI: 1.52, 8.22), and those who reached health facilities within 60 minutes (AOR=2.32; 95%CI: 1.21, 4.45).

**Conclusion:**

The study pinpointed age, knowledge, attitude toward cervical cancer screening, and accessibility to health facilities within a 60-minute radius as pivotal factors impacting cervical cancer screening uptake among reproductive-age women. These findings highlight the importance of targeted education, promoting positive attitudes, and enhancing healthcare accessibility to improve screening uptake and reduce the burden of cervical cancer.

## Introduction

Cervical cancer, a major global health issue, involves uncontrolled cell growth in the uterine cervix (1). It’s the leading cause of maternal illness and death worldwide, with 604,000 diagnoses and 342,000 deaths in 2020 (2, 3). According to Global Cancer Statistics 2020, it’s the fourth most common cause of cancer-related deaths among women, with a death occurring every two minutes, mostly in developing countries (4). In 2020, sub-Saharan Africa saw about 110,300 new cervical cancer cases, with cervical cancer being the second most common cancer among women in the region (5). In Ethiopia, it accounts for 10% of new cases in sub-Saharan Africa, making it the most prevalent cancer there and the second leading cause of death among reproductive-age women. Ethiopia reported approximately 7,445 new cases and 12,492 prevalent cases in 2020 (4).

Most cervical cancers are caused by persistent infection with high-risk HPV strains. While many HPV infections resolve on their own, persistent infections can lead to cervical pre-cancer, which, if untreated, may develop into cervical cancer over 10 to 20 years (6). Cervical cancer is preventable and curable if detected early, requiring more intensive prevention, detection, and treatment efforts than other gynecological cancers. Effective interventions in developed countries have reduced mortality rates (7). In August 2020, the WHO launched a strategy to eliminate cervical cancer, aiming to vaccinate 90% of eligible women against HPV, screen 70% at least twice, and treat 90% of those with positive results (6).

A study found that cervical cancer screening is the most effective prevention strategy, reducing deaths by 70% (8). Screening aims to detect and remove abnormal cells before cancer develops. Globally, methods include HPV testing, cytology (Pap test), and VIA (9). In resource-limited countries like Ethiopia, VIA is preferred and recommended for women, especially between ages 30-49. Evidence suggests that including younger women in screening and treatment strategies may also be beneficial (2, 6, 10). Studies show that cervical cancer affects patients in many ways, including societal discrimination, body image issues, sexual function impairment, income loss, financial strain, and employment challenges (11, 12). Despite these impacts, cervical cancer screening rates remain low, particularly in sub-Saharan Africa (13).

A 2021 review found cervical cancer screening uptake in sub-Saharan Africa was only 12.87% (3). In Ethiopia, the 2020 HSTP-I report indicated a screening rate of just 5%, with other studies showing a range from 2.5% to 38.7% (14–18), and another meta-analysis found 14.79% (19). In early 2022, screening rates at Mizan Tepi University, Gebretsadik Shewa General, and Bachuma Primary Hospitals were 0.5%, 0.3%, and 2.3%, respectively, all below the national average. Despite the low uptake, there’s a gap in understanding the factors affecting cervical cancer screening in Ethiopia. Therefore, this study aimed to assess the determinants of cervical cancer screening uptake among reproductive-age women at selected public hospitals in southwest Ethiopia.

## Methods

### Study design, setting, and period

A hospital-based unmatched case-control study was conducted at Mizan-Tepi University Teaching Hospital, Gebretsadik Shewa General Hospital, and Bachuma Primary Hospital in the southwest region of Ethiopia. This region, located 449 km from the capital city, consists of six zones. Among the 12 hospitals in the region, these three hospitals collectively serve over one million people.

Mizan Tepi University Teaching Hospital, located in Ethiopia’s southwest Bench Sheko Zone, serves communities in Bench Sheko, West Omo, Sheka, and Gambela regions. Established in 1986 as Mizan Teferi Hospital and integrated into Mizan Tepi University in 2016, it is situated 580 kilometers southwest of Addis Ababa. Gebretsadik Shewa General Hospital is situated in Bonga town, Kaffa Zone, and is 449 kilometers from Addis Ababa. Bachuma Primary Hospital, located in the West Omo Zone, was upgraded from a health center in 2017 and is approximately 660 kilometers from Addis Ababa and 180 kilometers from Bonga town. These hospitals provide cervical cancer screening services for reproductive-age women. The study was conducted from June 10 to August 25, 2022.

### Populations

The source population included all reproductive-age women seeking antenatal care, family planning services, and cervical cancer screening in the obstetrics and gynecology outpatient departments during the study period. Cases were women aged 15 to 49 who underwent cervical cancer screening, while controls were reproductive-age women visiting the hospitals for antenatal care or family planning services but not screened for cervical cancer. Exclusion criteria included women with known mental illness, those previously screened, those currently diagnosed and on follow-up, and women unable to provide written informed consent.

### Sample size determination

The study’s sample size was determined using Epi-info version 7.1, assuming a control-to-case ratio of 3, 80% power, and a 95% confidence level. The proportion of advanced age among controls (38.8%), with an odds ratio of 2.15, was based on a similar study in Ethiopia (20). To address non-response bias, an extra 10% was included, yielding a final sample size of around 420 participants (105 cases and 315 controls).

### Sampling procedure

**Figure 1:**
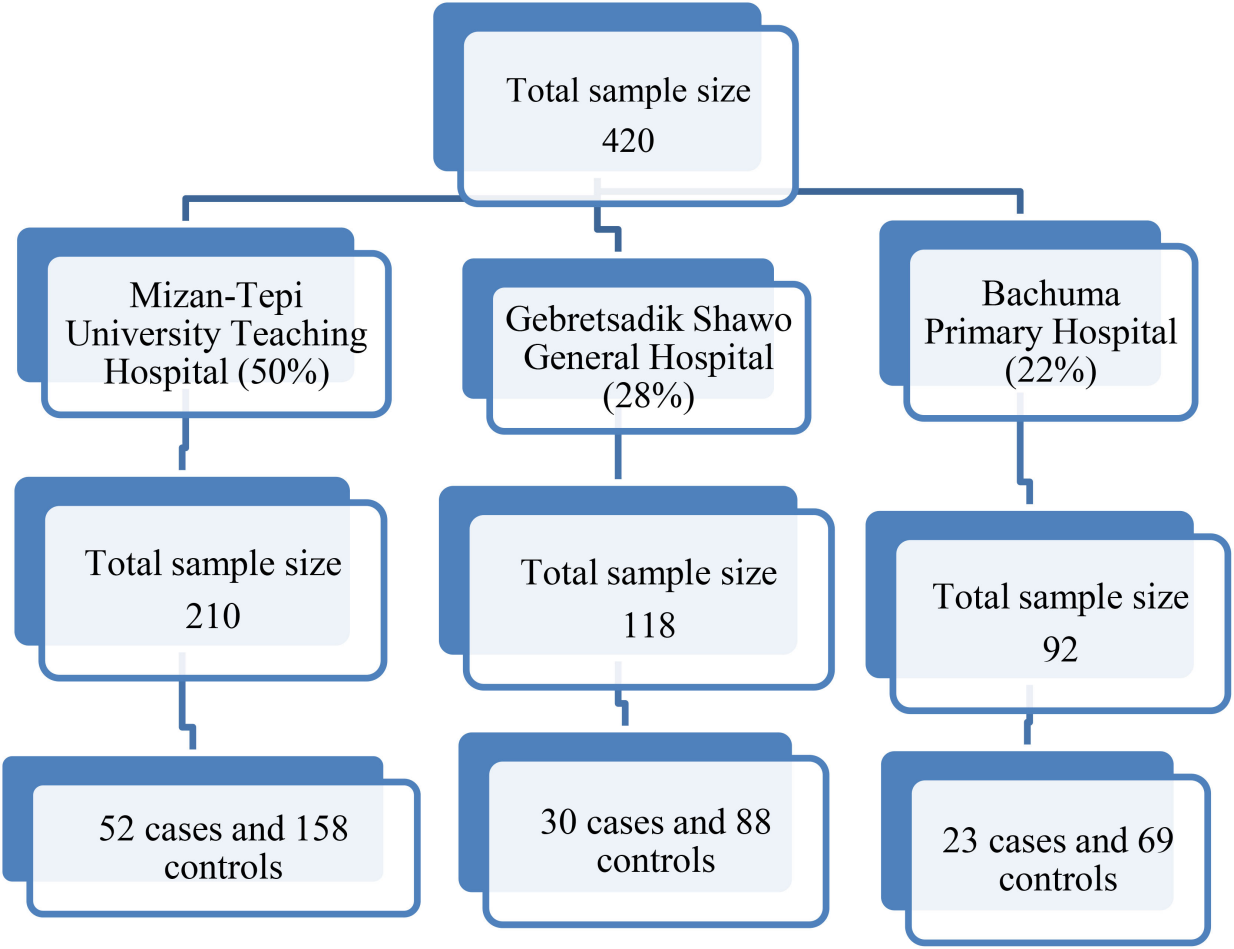
Sample size allocation for the three hospitals.

Mizan Tepi University Teaching Hospital, Gebretsadik Shewa General Hospital, and Bachuma Primary Hospital were purposively selected for their routine cervical cancer screening services. Sample allocation to these hospitals was based on the proportion of women screened monthly, as reported in the first quarter of 2022 (**Figure 1**). Cases were sampled consecutively until the required number was reached, with three controls selected for each case on the same day from the obstetrics and gynecology outpatient department using consecutive sampling.

### Study variables

The outcome variable was cervical cancer screening uptake, while the independent variables encompassed socio-demographic factors (age, education, marital status, occupation, income, religion, and residence), access to healthcare (travel time, transport means, cost of travel and perceived cost), knowledge of cervical cancer, knowledge and attitude toward screening, and medical and behavioral determinants (number of sexual partners, history of STDs, smoking, and HIV status).

### Operational definitions

Knowledge of cervical cancer: Knowledge levels were categorized based on Bloom’s cut-off points as follows: High level: Knowledge scores of 7 and 8 (80 – 100%). Moderate level: Knowledge scores of 5 and 6 (60 – 79.9%). Low level: Knowledge scores of 0 – 4 (<60%) (21).

Knowledgeable about cervical cancer screening: Knowledge levels were categorized based on Bloom’s cut-off points as follows: High level: Knowledge scores of 4 and 5 (80 – 100%). Moderate level: Knowledge score of 3 (60 – 79.9%). Low level: Knowledge scores of <3 (<60%) (21).

Attitude toward screening: The responses were categorized into three levels according to Bloom’s cut-off points: Positive attitude: Attitude scores ranging from 28 to 35 (80 – 100%). Neutral attitude: Attitude scores ranging from 21 to 27 (60 – 79.9%). Negative attitude: Attitude scores below 7 to 20 (<60%) (21).

Accessibility to the health facility: It was categorized as follows: Accessible: Time taken less than 60 minutes (distance <5 km). Not accessible: Time taken greater than 60 minutes (distance ≥5 km) (20).

### Data collection tools, and quality management

An interviewer-administered questionnaire, adapted from previous studies (20, 22), was utilized in the three selected hospitals. The questionnaire, originally prepared in English, was translated into Amharic by an experienced translator and back-translated into English by an independent translator to ensure consistency. In this study, attitude was evaluated using questions based on a Likert scale, with responses ranging from strongly disagree to strongly agree. The scoring system assigned: 5 for strongly agree, 4 for agree, 3 for neither agree nor disagree, 2 for disagree, and 1 for strongly disagree. Seven questions were utilized to assess attitude, and the responses were categorized into three levels based on Bloom’s cut-off points: 1 for negative, 2 for neutral, and 3 for positive. The minimum score was 7, and the maximum score was 35. Knowledge of cervical cancer was assessed through eight knowledge assessment questions, with each question having multiple responses. The responses were computed and recorded as either correct (1) or incorrect (0). The scores ranged from 0 to 8. Knowledge about cervical cancer screening was assessed using five knowledge assessment questions, each with multiple responses. The responses were computed and recorded as either correct (1) or incorrect (0), resulting in scores ranging from 0 to 5. Accessibility was measured by the total time taken to reach the health institution to access cervical cancer screening services in minutes when study subjects arrived on foot, or the distance in kilometers. Before actual data collection, the tools underwent pretesting with 21 study participants (5 cases &16 controls), which constituted 5% of the total sample size, to assess response accuracy, language clarity, and tool appropriateness. Six midwives, two from each hospital, underwent one-day training on data collection tools. Following training, these data collectors interviewed women visiting the hospitals for cervical cancer services after their appointments, while controls were interviewed after completing their visits. Each interviewed client received a sign on their card to prevent interview redundancy.

### Data processing and analysis

The data were cleaned, coded, and entered into EpiData version 4.6 before being exported to SPSS version 25 for analysis. Descriptive statistics, including frequencies and percentages, were computed and the results were presented in text, graphs, and tables. The model’s independent variables had an acceptable variance inflation factor (VIF < 2), indicating low multicollinearity. The model fit the data well, as confirmed by the Hosmer-Lemeshow test (p = 0.656). Candidate variables with a p-value below 0.25 in the bivariate regression were included in the multivariable logistic regression model to control for confounding effects. Predictors for cervical cancer screening uptake were identified based on a p-value < 0.05 and presented as adjusted odds ratios (AOR) with a 95% confidence interval.

## Results

### Socio-demographic characteristics

The study included 98 cases and 294 controls, achieving a 93.3% response rate. Among cases, 50% were aged 30-39 years, while 28.6% of controls. Education levels showed 13.2% of cases and 12.6% of controls with no formal education. Additionally, 12.3% of cases and 7.1% of controls were single, with 75.5% of cases and 71.8% of controls being married. Residence-wise, 81.6% of cases and 67.7% of controls lived in urban areas (**Table 1**).

**Table 1:** Factors associated with cervical cancer screening uptake among the study participants.

Variables	Categories	Cases	Controls	COR (95% CI)	AOR (95% CI)	*P-value*
		n (%)	n (%)			
Age (y)	20 – 29	19 (19)	140 (47.6)	1	1	
	30 – 39	49 (50)	84 (28.6)	4.31 (2.42, 7.83)**	3.94 (1.79, 8.63)	**0.001**
	40 – 49	30 (31)	70 (23.8)	3.22 (1.61, 6.13)*	3.54 (1.52, 8.22)	**0.003**
Religion	Orthodox	52 (53.1)	119 (40.5)	1	1	
	Muslim	17 (17.3)	37 (12.6)	1.05 (0.53, 2.24)	0.78 (0.31, 1.96)	0.601
	Protestant	24 (24.5)	111 (37.8)	0.49 (0.36, 0.92)	0.62 (0.29, 1.29)	0.199
	Catholic	5 (5.1)	27 (9.1)	0.42 (0.16, 1.24)	0.32 (0.09, 1.07)	0.065
Education	No formal education	13 (13.2)	37 (12.6)	1	1	
	Primary school	31 (31.6)	55 (18.7)	1.64 (0.72, 3.52)	1.31 (0.44, 3.91)	0.628
	Secondary school	27 (27.6)	71 (24.1)	1.11 (0.53, 2.34)	0.64 (0.21, 1.98)	0.440
	College and above	27 (27.6)	131 (44.6)	0.62 (0.35, 1.25)	0.39 (0.12, 1.25)	0.113
Marital status	Single	12 (12.3)	21 (7.1)	1	1	
	Married	74 (75.5)	211 (71.8)	0.63 (0.34, 1.35)	1.13 (0.43, 2.94)	0.802
	Divorced	7 (7.1)	35 (11.9)	0.44 (0.17, 1.02)	1.32 (0.33, 5.27)	0.696
	Widowed	5 (5.1)	27 (9.2)	0.38 (0.09-1.06)	1.33 (0.27, 6.59)	0.726
Residence	Rural	18 (18.4)	95 (32.3)	1	1	
	Urban	80 (81.6)	199 (67.7)	2.12 (1.26, 3.71)*	0.67 (0.29, 1.55)	0.349
Occupation	Government employee	29 (29.6)	99 (33.7)	1	1	
	Private employee	37 (37.8)	135 (45.9)	0.94 (0.51, 1.62)	0.74 (0.33, 1.69)	0.480
	Housewife	30 (30.6)	48 (16.3)	2.10 (1.23, 3.94)*	1.79 (0.71, 4.56)	0.220
	Others^#^	2 (2)	12 (4.1)	0.57 (0.12, 2.69)	0.75 (0.08, 6.76)	0.798
Travel time to healthcare facilities	≥60 minutes	49 (50)	194 (66)	1	1	
	<60 minutes	49 (50)	100 (34)	1.94 (1.22, 3.08)*	2.32 (1.21, 4.45)	**0.011**
Cervical cancer knowledge	Low level	8 (8.2)	149 (50.7)	1	1	
	Moderate level	10 (10.2)	32 (10.9)	13.2 (6.10, 28.4)*	1.66 (0.40, 6.85)	0.483
	High level	80 (81.6)	113 (38.4)	5.82 (2.15, 15.9)*	1.64 (0.45, 6.00)	0.454
Knowledge of screening	Low level	10 (10.2)	182 (61.9)	1	1	
	Moderate level	6 (6.1)	17 (5.8)	15.7 (7.81, 31.7)*	3.88 (0.89, 16.9)	0.071
	High level	82 (83.7)	95 (32.3)	6.43 (2.08, 19.8)**	6.23 (1.96, 19.8)	**0.002**
Attitude towards screening	Negative	12 (12.3)	169 (57.5)	1	1	
	Neutral	16 (16.3)	55 (18.7)	14.1 (7.22, 27.6)*	1.71 (0.65, 4.49)	0.278
	Positive	70 (71.4)	70 (23.8)	4.18 (1.84, 9.24)**	6.12 (2.40, 15.6)	**<0.001**

N.B, AOR, Adjusted odds ratio; CI, Confidence interval; COR, Crude odds ratio; n, frequency; ^*^p-value<0.05; ^**^p-value<0.001; Others^#^: Merchant, daily labor and student.

The bold values used to show statistical significance.

### Access to healthcare facilities

**Figure 2:**
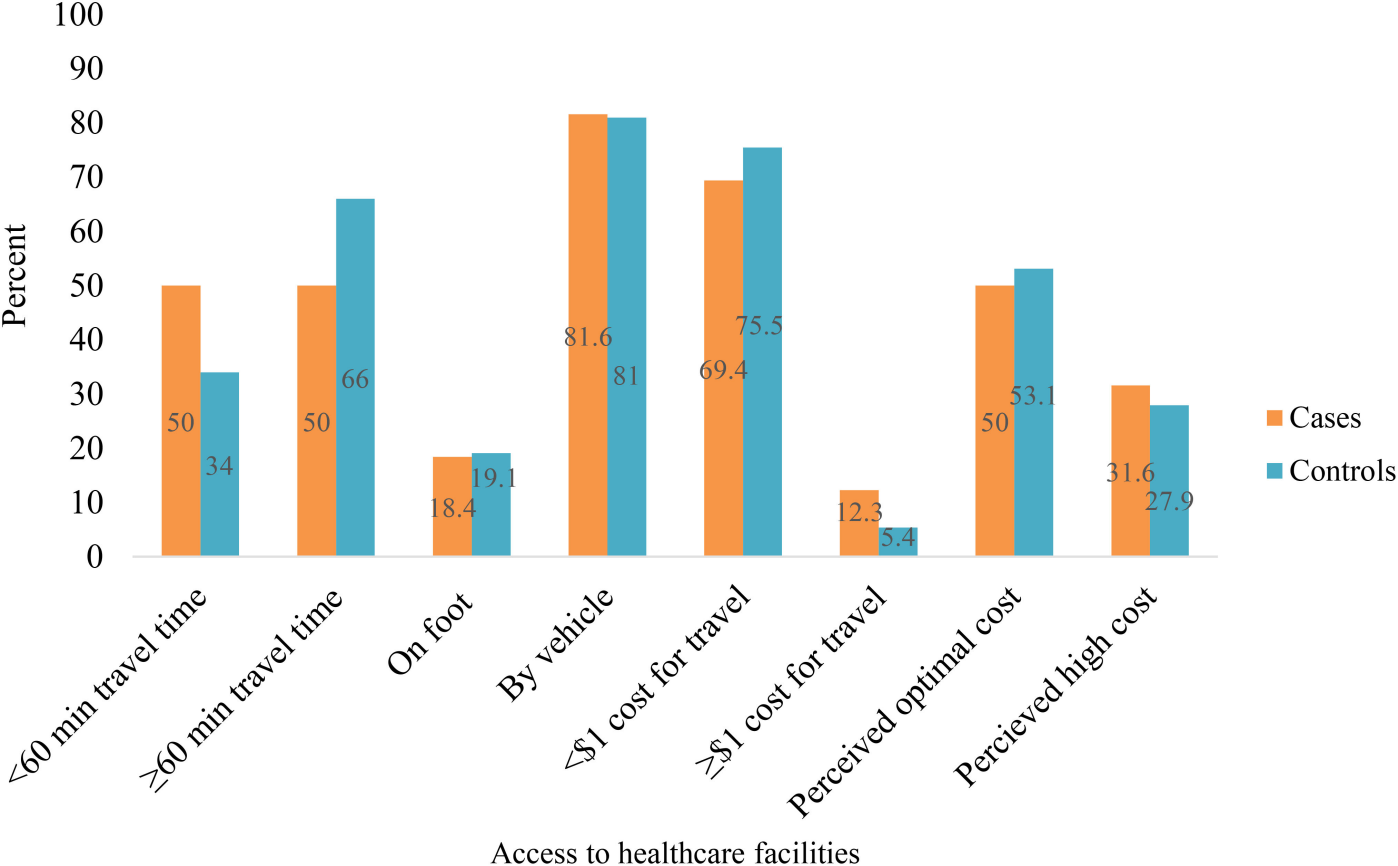
Access to healthcare facilities among participants.

Fifty percent of cases reached the health facility within 60 minutes, compared to 34% of controls. Among cases, 81.6% used public transportation, while 18.6% walked on foot (**Figure 2**).

### Medical and behavioral characteristics

Thirteen point three percent of cases and 15.7% of controls reported having multiple sexual partners. Regarding smoking status, 4.1% of cases and 9.5% of controls were smokers. Among cases, 5.1% had a history of sexually transmitted diseases (STDs), compared to 4.4% of controls. Additionally, 8.2% of cases and 4.4% of controls tested positive for HIV (**Supplementary Figure S1**).

### Knowledge of cervical cancer

Among the cases, 87.8% were aware of cervical cancer, compared to 50.7% of controls. Among those aware of cervical cancer, 75.6% of cases and 65.1% of controls recognized viruses as the cause. For symptoms, 59.3% of cases and 27.9% of controls identified vaginal bleeding. Overall, 81.6% of cases had high knowledge, compared to 38.4% of controls (**Supplementary Table S1**).

### Knowledge about cervical cancer screening

Ninety cases (91.8%) and 39.5% of controls were aware of cervical cancer screening. Public media was the primary source of information for 60% of cases and 55.2% of controls. Overall, 83.7% of cases and 32% of controls demonstrated high knowledge about cervical cancer screening (**Supplementary Table S2**).

### Attitude towards cervical cancer screening

Seventy-one cases (72.4%) and 46.2% of controls agreed that cervical cancer is deadlier than other cancers. Eighty-eight cases (85.6%) and 36% of controls were willing to undergo screening. Overall, 71% of cases and 24% of controls had a positive attitude toward cervical cancer screening (**Supplementary Table S3**).

### Factors associated with cervical cancer screening uptake

After adjusting for confounding variables, women aged 30–39 (AOR=3.94; 95% CI: 1.79, 8.63) and those aged 40–49 (AOR=3.54; 95% CI: 1.52, 8.22) were more likely to undergo cervical cancer screening. Additionally, women with a high knowledge of screening (AOR=6.23; 95% CI: 1.96, 19.79), a positive attitude toward screening (AOR=6.12; 95% CI: 2.40, 15.58), and those who reached health facilities within 60 minutes (AOR=2.32; 95% CI: 1.21, 4.45) were also more likely to participate in screening (**Table 1**).

## Discussion

Cervical cancer screening is vital for the early detection and treatment of precancerous lesions (23). This study sought to identify the factors influencing cervical cancer screening uptake among women of reproductive age in southwest Ethiopia. It found that key determinants include women’s age, their level of knowledge about cervical cancer screening, their attitude towards screening, and the time required to reach health facilities.

The study revealed that women aged 30 – 39 and 40 – 49 were respectively 3.9 and 3.5 times more likely to undergo cervical cancer screening compared to those aged 20 – 29. This finding aligns with similar studies conducted in various Ethiopian regions like Ambo, Diredawa, Mekele, and Finoteselam, which also observed higher screening rates among older age groups (16, 20, 24, 25). Older women may be more inclined to undergo cervical cancer screening due to a perceived higher risk associated with their age. Moreover, increased exposure to healthcare facilities as women age could contribute to higher screening rates among older age groups. Indeed, a study conducted in India revealed that younger women were more inclined to undergo cervical cancer screening compared to older age groups (26). This discrepancy could stem from differences in information availability and variations in the study participants’ characteristics.

This study found that having a high knowledge of cervical cancer screening was a significant predictor of uptake. Women with a high of knowledge screening were 6 times more likely to undergo cervical cancer screening compared to those with low knowledge of screening. This finding aligns with studies conducted in various regions of Ethiopia, such as Mekele, Ambo, Addis Ababa, Jimma, and Adigrat. These studies also demonstrated that women with a high knowledge of cervical cancer screening were more inclined to utilize screening services compared to those with lower knowledge of screening (20, 22, 25, 27, 28). Studies conducted worldwide have consistently shown that women with a high knowledge of cervical cancer screening are more likely to utilize screening services compared to those with low knowledge (29, 30). This trend suggests that informed women may exhibit higher health-seeking behavior and intentions to undergo screening. Access to various sources of information, such as media, may contribute to their increased awareness and motivation to seek healthcare services, including cervical cancer screening. Consequently, women with a good understanding of cervical cancer are more likely to uptake screening services than those with poor knowledge levels.

A positive attitude toward cervical cancer screening significantly increased the likelihood of uptake, as women with a positive attitude were 6 times more likely to undergo screening compared to those with a negative attitude toward screening. The findings of this study align with previous research conducted in various regions of Ethiopia (20, 31, 32). Similarly, a study conducted in Gondar, Ethiopia, found that women with a favorable attitude were more inclined to utilize cervical cancer screening services compared to those with an unfavorable attitude (33). Women with a positive attitude towards screening may possess a stronger sense of self-care and respect for their well-being, potentially leading to better health-seeking behaviors. Moreover, this positive attitude may drive them towards seeking a healthier lifestyle, thus making them more likely to engage in preventive healthcare practices such as cervical cancer screening.

In this study, the time taken to reach health facilities emerged as a predictor of cervical cancer screening uptake. Women who reached health facilities within 60 minutes had 2 times higher odds of being screened compared to their counterparts. This study aligns with research conducted in Uganda and South Africa, indicating that women who had access to health facilities offering cervical cancer screening services were more likely to undergo screening compared to those who lacked such accessibility (30, 34). The evidence suggests that proximity to health facilities plays a crucial role in facilitating cervical cancer screening uptake. Women residing closer to health facilities have greater opportunities to access various services and information from healthcare professionals, thereby increasing their likelihood of undergoing screening. Additionally, accessibility is influenced by travel costs, with women living nearer to health facilities incurring lower expenses. This affordability may enhance their willingness to seek healthcare, consequently leading to a higher uptake of cervical cancer screening.

### Limitations of the study

As a hospital-based study, the results may not apply to the general population. The small sample size limits the ability to generalize the findings to a larger population because it decreases the statistical power and reliability of the results. Recall bias and social desirability bias could affect the findings, particularly for variables like STI history and smoking status. Additionally, establishing a clear temporal relationship between factors such as attitude, knowledge, and cervical cancer screening uptake is challenging.

## Conclusion

The research identified age, knowledge, and attitude regarding cervical cancer screening, along with proximity to health facilities within a 60-minute radius, as key factors influencing cervical cancer screening uptake among women of reproductive age. These findings highlight the importance of targeted education, promoting positive attitudes, and enhancing healthcare accessibility to improve screening uptake and reduce the burden of cervical cancer.

## Data Availability

The raw data supporting the conclusions of this article will be made available by the authors, upon reasonable request.
