# Supplementary material for: Determinants of cervical cancer screening uptake among reproductive-age women in southwest Ethiopia: a case-control study
Source: Front Oncol. 2024 Oct 23;14:1424810. doi: 10.3389/fonc.2024.1424810 (PMC11537959; doi:10.3389/fonc.2024.1424810)
Supplement: Supplementary file 1 [file DataSheet1.docx]

**Supplementary files**

Supplementary Table 1: Knowledge of cervical cancer among study participants.

| Variables | Categories | Cases, n (%) | Controls, n (%) |
| --- | --- | --- | --- |
| Have you heard of cervical cancer? | Yes | 86 (87.8) | 149 (50.7) |
|  | No | 12 (12.2) | 145 (49.3) |
| Where did you hear about it? | Health professionals | 26 (30.2) | 43 (28.9) |
|  | Public media (TV, Radio) | 48 (55.8) | 83 (55.7) |
|  | Neighborhoods | 12 (14.0) | 23 (15.4) |
| What causes cervical cancer? | Virus | 65 (75.6) | 97 (65.1) |
|  | Bacteria | 13 (15.1) | 45 (30.2) |
|  | Fungus | 6 (7.0) | 5 (3.4) |
|  | Protozoa | 2 (2.3) | 2 (1.3) |
| What are the signs and symptoms of cervical cancer? | Vaginal bleeding | 51 (59.3) | 82 (55.0) |
|  | Foul smelling discharge | 21 (24.4) | 32 (21.5) |
|  | Post coital bleeding | 5 (5.8) | 26 (17.5) |
|  | I don’t know | 9 (10.5) | 9 (6.0) |
| What are the risk factors for cervical cancer? | HPV | 56 (65.1) | 77 (51.7) |
|  | Multiple sexual partners | 16 (18.6) | 50 (33.6) |
|  | Early sexual intercourse | 8 (9.3) | 3 (2.0) |
|  | Multiple children | 1 (1.2) | 6 (4.0) |
|  | Long term use of oral contraceptive pills | 0 (0.0) | 5 (3.4) |
|  | HIV/AIDS | 3 (3.5) | 7 (4.7) |
|  | I don’t know | 2 (2.3) | 1 (0.7) |
| Who is at risk of cervical cancer? | >50 years | 43 (50.0) | 66 (44.3) |
|  | Reproductive age group | 28 (32.6) | 68 (45.6) |
|  | Both | 15 (17.4) | 15 (10.1) |
| What are the prevention methods for cervical cancer? | HPV vaccination | 64 (74.4) | 73 (49.0) |
|  | Avoiding multiple sexual partner | 13 (15.1) | 52 (34.9) |
|  | No early age sexual intercourse | 5 (5.8) | 20 (13.4) |
|  | Not having multiple children | 4 (4.7) | 4 (2.7) |
| Is cervical cancer curable? | Yes | 83 (96.5) | 102 (68.5) |
|  | No | 2 (2.3) | 41 (27.5) |
|  | I do not know | 1 (1.2) | 6 (4.0) |
| Overall knowledge of cervical cancer | High level | 80 (81.6) | 113 (38.4) |
|  | Moderate level | 8 (8.2) | 32 (10.9) |
|  | Low level | 10 (10.2) | 149 (50.7) |

Supplementary Table 2: Knowledge of cervical cancer screening among study participants.

| Variables | Categories | Cases, n (%) | Controls, n (%) |
| --- | --- | --- | --- |
| Heard about screening | Yes | 90 (91.8) | 116 (39.5) |
|  | No | 8 (8.2) | 178 (60.5) |
| Source of information | Health professionals | 25 (27.8) | 36 (31.0) |
|  | Public medias | 54 (60.0) | 64 (55.2) |
|  | Neighborhood | 11 (12.2) | 16 (13.8) |
| Screening duration | Yearly | 22 (24.4) | 60 (51.7) |
|  | Every three years | 58 (64.5) | 52 (44.8) |
|  | Every five years | 10 (11.1) | 4 (3.5) |
| Screening target | >25 years | 51 (56.7) | 62 (53.5) |
|  | Prostitutes | 27 (30.0) | 40 (34.5) |
|  | Elderly women | 10 (11.1) | 9 (7.7) |
|  | I don’t know | 2 (2.2) | 5 (4.3) |
| Screening is expensive | Yes | 53 (58.9) | 51 (44.0) |
|  | No | 25 (27.8) | 49 (42.2) |
|  | I don’t know | 12 (13.3) | 16 (13.8) |
| Overall knowledge of cervical screening | High level | 82 (83.7) | 95 (32.3) |
|  | Moderate level | 6 (6.1) | 17 (5.8) |
|  | Low level | 10 (10.2) | 182 (61.9) |

Supplementary Table 3: Attitude towards cervical cancer screening among study participants.

| Variables | Responses | Cases, n (%) | Controls, n (%) |
| --- | --- | --- | --- |
| Cervical cancer is more deadly than other cancers | Strongly disagree | 3 (3.1) | 10 (3.4) |
|  | Disagree | 14 (14.3) | 50 (17.0) |
|  | Neutral | 10 (10.2) | 98 (33.3) |
|  | Agree | 41 (41.8) | 105 (35.7) |
|  | Strongly agree | 30 (30.6) | 31 (10.5) |
| All women are at risk | Strongly disagree | 4 (4.1) | 7 (2.4) |
|  | Disagree | 9 (9.2) | 63 (21.4) |
|  | Neutral | 10 (10.2) | 94 (32.0) |
|  | Agree | 44 (44.9) | 102 (34.7) |
|  | Strongly agree | 31 (31.6) | 28 (9.5) |
| Cervical cancer is transmissible | Strongly disagree | 4 (4.1) | 5 (1.7) |
|  | Disagree | 7 (7.1) | 84 (28.6) |
|  | Neutral | 9 (9.2) | 89 (30.3) |
|  | Agree | 50 (51.0) | 82 (27.9) |
|  | Strongly agree | 28 (28.6) | 34 (11.6) |
| Screening prevents cervical cancer | Strongly disagree | 6 (6.1) | 5 (1.7) |
|  | Disagree | 8 (8.2) | 88 (29.9) |
|  | Neutral | 4 (4.1) | 85 (28.9) |
|  | Agree | 45 (45.9) | 76 (25.9) |
|  | Strongly agree | 35 (35.7) | 40 (13.6) |
| Screening is safe | Strongly disagree | 2 (2.0) | 5 (1.7) |
|  | Disagree | 9 (9.2) | 107 (36.4) |
|  | Neutral | 4 (4.1) | 83 (28.3) |
|  | Agree | 51 (52) | 73 (24.8) |
|  | Strongly agree | 32 (32.7) | 26 (8.8) |
| Screening is affordable | Strongly disagree | 3 (3.1) | 1 (0.3) |
|  | Disagree | 8 (8.2) | 101 (34.4) |
|  | Neutral | 6 (6.1) | 106 (36.1) |
|  | Agree | 45 (45.9) | 63 (21.4) |
|  | Strongly agree | 36 (36.7) | 23 (7.8) |
| Volunteered for cervical screening | Strongly disagree | 2 (2.0) | 7 (2.4) |
|  | Disagree | 9 (9.2) | 91 (31.0) |
|  | Neutral | 9 (9.2) | 90 (30.6) |
|  | Agree | 43 (43.9) | 83 (28.2) |
|  | Strongly agree | 35 (35.7) | 23 (7.8) |
| Overall attitude toward cervical screening | Positive | 70 (71.4) | 70 (23.8) |
|  | Neutral | 16 (16.3) | 55 (18.7) |
|  | Negative | 12 (12.3) | 169 (57.5) |

Supplementary Figure 1: Medical and behavioral characteristics of the study participants.
